# Epidemiology of myocardial infarction in Korea: hospitalization incidence, prevalence, and mortality

**DOI:** 10.4178/epih.e2022057

**Published:** 2022-07-12

**Authors:** Rock Bum Kim, Jang-Rak Kim, Jin Yong Hwang

**Affiliations:** 1 Department of Preventive Medicine and Institute of Health Sciences, Gyeongsang National University College of Medicine, Jinju, Korea; 2 Department of Internal Medicine, Gyeongsang National University College of Medicine and Gyeongsang National University Hospital, Jinju, Korea

**Keywords:** Myocardial infarction, Epidemiology, Incidence, Prevalence, Mortality

## Abstract

Few studies have comprehensively presented epidemiological indicators of myocardial infarction in Korea. However, multiple published articles and open-source secondary data on the epidemiology of myocardial infarction are now available. This review summarized the hospitalization incidence, prevalence, and mortality rate of myocardial infarction in Korea using articles and open-source data from the Health Insurance Service and the Department of Statistics, surveys of sample populations, registries of patients, and other sources. The epidemiological indicators of myocardial infarction were compared between Korea and other high-income countries. The incidence of hospitalization due to myocardial infarction in Korea was 43.2 cases per 100,000 population in 2016 and has consistently increased since 2011. It was 2.4 times higher among men than among women. The estimated prevalence among adults over 30 years of age ranged from 0.34% to 0.70% in 2020; it was higher among men and increased with age. The mortality in 2020, which was 19.3 per 100,000 population in 2020, remained relatively stable in recent years. Mortality was higher among men than among women. Based on representative inpatient registry data, the proportion of ST-elevated myocardial infarction decreased until recently, and the median time from symptom onset to hospital arrival was approximately 2 hours and 30 minutes. The hospitalization incidence, prevalence, and mortality rate of myocardial infarction were lower in Korea than in other countries, although there was an increasing trend. Comprehensive national-level support and surveillance systems are needed to routinely collect accurate epidemiological indicators.

## GRAPHICAL ABSTRACT

[Fig f7-epih-44-e2022057]

## INTRODUCTION

Epidemiological indicators, such as the hospitalization incidence, prevalence, and mortality rate, provide clinicians with information on the scale and historical trends of diseases. These data can be used to develop and support healthcare policies and provide important data for assessing these policies in the future. Given the considerable effort, financial resources, and time required to collect them, few epidemiological indicators related to myocardial infarction in Korea are available. However, in recent years, secondary sources of data from large-scale health insurance claims databases and survey data have become available, making it possible to indirectly calculate epidemiological indicators for myocardial infarction.

This paper presents a review and analysis of the hospitalization incidence, prevalence, and mortality rate (including case-fatality) of myocardial infarction in Korea using published articles and open-source secondary data from the Healthcare Bigdata Hub [1], Korean Statistical Information Service of Statistics Korea [2], Korea National Health and Nutrition Examination Survey (KNHANES) [3], patient registry data, and other sources. This paper described the annual trends, strengths, and weaknesses of these indirect epidemiological indicators for interpretation. In addition, it analyzed the same indicators from other high-income countries by reviewing and comparing open-source secondary data at the website, and although the data were not methodologically controlled enough to make a perfect comparison, the comparisons in this study may be helpful for understanding the significance of the indicators from Korea.

## SOURCES FOR REVIEWING EPIDEMIOLOGICAL INDICATORS

### Sources for incidence in Korea

A large cohort study is required to accurately estimate the incidence of acute myocardial infarction. However, it is difficult to estimate the incidence due to the considerable funding and time required to undertake such research. Therefore, research on the incidence of acute myocardial infarction tends to focus on secondary data.

One study from Korea [4] estimated the incidence using claims data from the National Health Insurance Service. In that study, new cases of acute myocardial infarction were defined as patients hospitalized for more than 1 day with an International Classification of Disease, 10th edition (ICD-10), code of I21 due to its increased diagnostic accuracy and who received a myocardial enzyme test, percutaneous coronary intervention (PCI), and antiplatelet therapy and did not have the above-mentioned disease or undergo these procedures within 5 years. Patients who died within 24 hours of hospitalization were excluded.

### Sources for incidence in other countries

The incidence of myocardial infarction in other countries was reviewed or re-analyzed using published articles or open-source statistical data available online. When there were several sources, we reviewed the most recent sources that included as large patients as possible or cohort studies.

One study from Japan [5] included patients diagnosed with acute myocardial infarction based on the universal definition from the European Society of Cardiology/American College of Cardiology committee in 2000 [6]. The study calculated the incidence rate based on the population of Tokyo for 51,639 patients admitted to 72 hospitals in the Tokyo area, which included about 95% of total patients in the Tokyo area.

For the United States, we conducted a web search of the Minnesota Public Health Data Access Portal (https://data.web.health.state.mn.us/mci). The data on the website were constructed and managed by the Minnesota Department of Health. It contained statistical data on several diseases and conditions including heart attack (International Classification of Diseases, Ninth Revision, Clinical Modification [ICD-9-CM] code 410 or ICD-10 code I21 or I22, equal to acute or subsequent myocardial infarction). The data for hospitalization or emergency department patients (aged 35 years or older) with heart attack were extracted from Minnesota hospital discharge data representing 99.4% of all hospitals in the state of Minnesota.

For the United Kingdom, we searched the Hospital Episode Statistics (HES) database (https://digital.nhs.uk/data-and-information/data-tools-and-services/data-services/hospital-episode-statistics) managed by the National Health Service’s digital organization. The HES database contains patient details for all admissions, including emergency department and outpatient admissions, at National Health Service hospitals in England. From the website, we downloaded and analyzed raw data from 2018/2019 on the number of acute myocardial infarction (ICD-10 codes I21 or I22) patients hospitalized in England.

In Sweden, beginning in the 1960s, the National Board of Health and Welfare collected information on inpatients at public hospitals with the National Patient Register (NPR). In the present day, the NPR includes all inpatient care, outpatient care including outpatient surgery, and psychiatric care from both private and public caregivers. The statistical data from the NPR (number of incident cases, incidence rate, etc.) for acute myocardial infarction patients (aged 20 years and older with ICD-10 codes of I21 or I22) in all regions of Sweden are provided via the NPR website (https://sdb.socialstyrelsen.se/if_hji/val_eng.aspx).

### Sources for prevalence in Korea

Since the Health Insurance Review and Assessment Service (HIRA) publishes disease statistics [1] online (http://opendata.hira.or.kr/op/opc/olapMfrnIntrsIlnsInfo.do) every year and most cases of myocardial infarction require inpatient or outpatient treatment, it is possible to estimate the prevalence of myocardial infarction in Korea based on the number of patients who had an ICD-10 diagnostic code of I21 or I22 divided by the number of people (aged 30 years and older) in Korea. HIRA data capture national health insurance data related to acute myocardial infarction patients who received outpatient or inpatient treatment at all hospitals in Korea.

In Korea, the KNHANES randomly samples approximately 4,800 households every year. In the survey, a myocardial infarction diagnosis is determined by asking a single question (“Do you currently have a disease related to myocardial infarction?”) to all adult respondents over the age of 30 years old [3]. We analyzed the prevalence of myocardial infarction from online survey data (https://knhanes.kdca.go.kr/knhanes/main.do).

### Sources for prevalence in other countries

In the United States, the National Health and Nutrition Examination Survey (NHANES) is completed by a nationally representative sample of about 5,000 people every year. The prevalence is analyzed based on data for all respondents (aged 20 years and older) who responded “yes” to the question “Has a doctor or other health professional ever told you that you had a heart attack or MI [myocardial infarction]?” [7]. The Center for Disease Control of the United States provides the dataset from the survey on its website (https://wwwn.cdc.gov/nchs/nhanes/Default.aspx).

In the United Kingdom, the British Heart Foundation hosts open-source datasets with heart attack statistics (mortality, incidence, prevalence, treatments, costs, and risk factors) for the total population of the United Kingdom on its website (https://www.bhf.org.uk/what-we-do/our-research/heart-statistics/heart-statistics-publications/cardiovascular-disease-statistics-2020). The statistics are calculated using data from The Health Improvement Network, an electronic medical records database containing systematically recorded anonymous data on more than 3 million individuals (6% of the United Kingdom population) currently registered at participating primary care practices in the United Kingdom [8].

### Sources for mortality

In most countries, statistics on the number and causes of deaths are reported annually. In addition, the World Health Organization (WHO) shares raw mortality data on its website (https://www.who.int/data/data-collection-tools/who-mortality-database) for analyzing mortality rates in various countries around the world. Based on the WHO mortality database, the Organization for Economic Cooperation and Development (OECD) calculates mortality rates (including myocardial infarction, with ICD-10 codes of I21 or I22) for its 25 member countries and shares the data on its website for easy comparison (https://stats.oecd.org/Index.aspx).

### Sources for registries in Korea

Large-scale secondary data are useful for identifying the overall incidence and prevalence of myocardial infarction in Korea but lack detailed information on the type of event, the patient’s overall health status, and treatment outcomes. Therefore, it is necessary to analyze patient registration data to determine this information for the population of Korea.

Currently, patient registration data for Korea are collected by the Korea Acute Myocardial Infarction Registry (KAMIR) managed by the Myocardial Infarction Research Association [9] and the Korean Registry of Acute Myocardial Infarction (KRAMI) managed by the Regional Cardiocerebrovascular Disease Centers [10], in addition to the National Emergency Department Information Network (NEDIS) [11], which collects emergency room treatment data on patients with myocardial infarction who visit the National Emergency Medical Center. The KAMIR and KRAMI are registries maintained by cardiologists that include accurate diagnosis and treatment information for patients with myocardial infarction and follow-up information from up to 1 year after discharge. However, these registries are limited since the KAMIR and KRAMI only collect data from 20 participating and 14 participating hospitals, respectively, while the NEDIS collects information on myocardial infarction patients at all emergency rooms in Korea. Since its focus is on the pre-hospital stage, however, the NEDIS does not include information on diagnosis, treatment, and post-discharge at the hospital stage. We examined published data and data from registries on the type of myocardial infarction, namely ST-segment elevation myocardial infarction (STEMI) or non-ST-segment elevation myocardial infarction (NSTEMI), the time from symptom onset to hospital arrival, and the mortality rate after hospitalization.

### Sources for registries in other countries

Some countries also operate registration systems for patients with myocardial infarction (patients who had confirmed symptoms, who completed clinical tests, and who had undergone procedures in a hospital). However, since the data from these registries are not easily accessible, the characteristics of myocardial infarction patients in other countries were instead confirmed using published articles. Using a Kim et al. [10] article, we reviewed myocardial infarction hospitalization rates and 1-year myocardial infarction mortality rates of patients in registries from Japan (Japanese Acute Myocardial Infarction Registry, JAMIR), China (China Acute Myocardial Infarction), Sweden (Swedish Heart Intensive Care Admissions, SWEDEHEART), Switzerland (Acute Myocardial Infarction in Switzerland), and the United States (Acute Coronary Treatment and Intervention Outcomes Network Registry, ACTION-Registry).

## INCIDENCE OF HOSPITALIZATION FROM ACUTE MYOCARDIAL INFARCTION

### Hospitalized incidence and trends among the total population

The age-standardized incidence of hospitalization from myocardial infarction in Korea per 100,000 population in 2016 was calculated to be 43.2 cases [4], meaning that, in 2016 alone, there were 25,531 cases, corresponding to a rate of approximately 1 new case every 20 minutes, which is approximately 70 cases per day.

**Figure 1.**
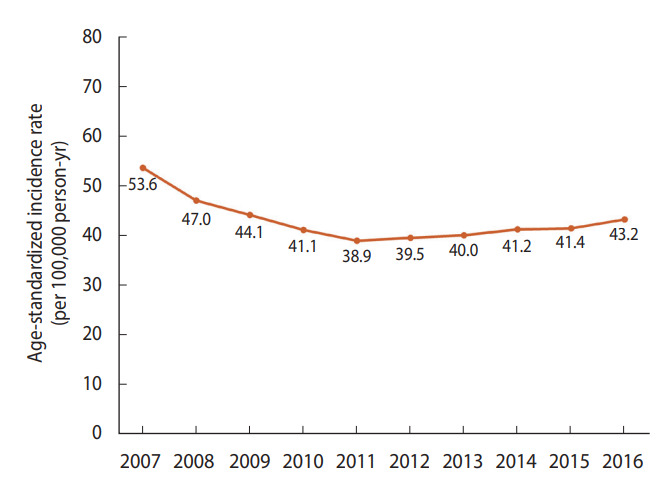
Age-standardized incidence of hospitalization for acute myocardial infarction in Korea between 2007 and 2016. Source: Adapted from Kim RB et al. J Korean Med Sci 2019;34:e322 [4].

The incidence of hospitalization from acute myocardial infarction per 100,000 population decreased steadily from 53.6 cases in 2007 to 38.9 in 2011 and began to increase after 2011, reaching 43.2 in 2016 (Figure 1). However, this increase was likely not a direct result of an increase in the incidence of the disease. Rather, patients who would have been diagnosed with unstable angina in the past were instead diagnosed with non-ST segment myocardial infarction after the introduction of the high-sensitivity troponin assay.

### Incidence by gender and age group

Analysis of claims data from the National Health Insurance Service revealed that the incidence of acute myocardial infarction in Korea per 100,000 population in 2016 totaled 70.5 cases for men and 29.3 cases per 100,000 for women, indicating that the incidence was 2.4 times higher among men.

By age group, those aged 80 years or older had the highest incidence rate of myocardial infarction per 100,000 population, at 321.4 cases, followed by 197.4 cases among those aged 70 years to 79 years, 117.7 cases among those aged 60 years to 69 years, 66.4 cases among those aged 50 years to 59 years, 29.3 cases among those aged 40 years to 49 years, 6.9 cases among those aged 30 years to 39 years, and 0.3 cases among those aged 29 years and under.

### Incidence by region

**Figure 2.**
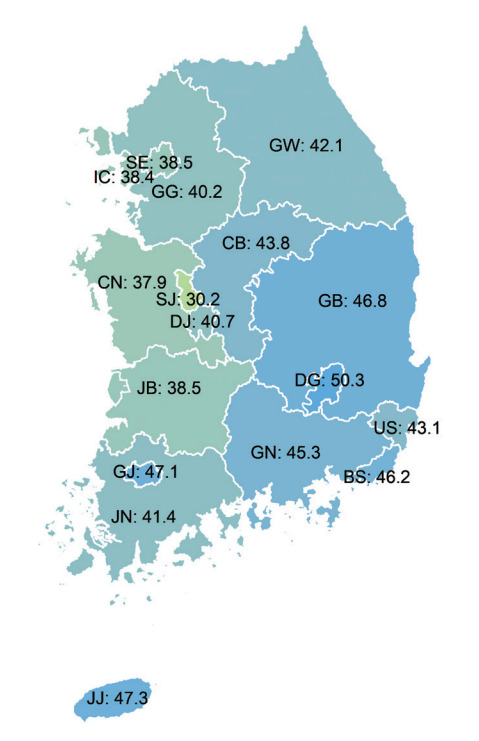
Average annual age-standardized incidence of hospitalization for acute myocardial infarction across 17 regions over 10 years. SE, Seoul; BS, Busan; DG, Daegu; IC, Incheon; GJ, Gwangju; DJ, Daejeon; US, Ulsan; SJ, Sejong; GG, Gyeonggi; GW, Gangwon; CB, Chungbuk; CN, Chungnam; JB, Jeonbuk; JN, Jeonnam; GB, Gyeongbuk; GN, Gyeongnam; JJ, Jeju. Source: Adapted from Kim RB et al. J Korean Med Sci 2019;34:e322 [4].

Claims data collected by the National Health Insurance Service includes patients’ addresses, thereby enabling comparisons by region in Korea. Of the 17 regions in Korea, Daegu was found to have the highest average age-standardized annual incidence per 100,000 population over 10 years (2007-2016) at 50.3 cases, followed by Jeju-do (47.3 cases) and Gwangju (47.1 cases). Sejong-si had the lowest incidence rate at 30.2 cases, followed by Chungcheongnam-do and Incheon, at 37.9 cases and 38.4 cases, respectively (Figure 2).

### Incidence and trends in other countries

Table 1 is showed the incidence and trends in Korea, Japan, United States, United Kingdom, and Sweden. In Japan, the age-standardized incidence rate per 100,000 population in 2016 was 40.7 cases (67.1 cases for men, 17.1 cases for women). There were no significant changes in the trend of incidence in the 11 years since 2006 (41.3 cases in total) [5].

In a community cohort study from the state of Minnesota in the United States, the age-standardized incidence per 100,000 population in 2012 was 84 cases for type 1 myocardial infarction and 78 cases for type 2 myocardial infarction. The incidence rate continued to decrease (by 3.3% per year) from 2003 to 2012 for both type 1 (202 cases to 84 cases) and type 2 (130 cases to 84 cases) myocardial infarction [12]. Furthermore, according to Minnesota Public Health Data, the incidence of myocardial infarction requiring hospitalization per 100,000 population was 261 cases in 2018 and had been decreasing steadily since 2000, which had a rate of 429 cases [13]. By gender, the age-standardized incidence rate per 100,000 population in 2018 was 358.0 cases for men and 176.0 cases for women.

In the Atherosclerosis Risk in Communities study, which was conducted in the United States, data from 2005 to 2014 showed that 1 new myocardial infarction occurred every 40 seconds, which translates to as many as 605,000 cases (190 cases per 100,000 population) every year. However, this figure has gradually decreased in recent years [7].

In the 2020 Heart and Circulatory Disease Statistics report from the United Kingdom, the number of patients with acute myocardial infarction in 2018/2019 was 100,963 (151.5 cases per 100,000 population), and 65,699 (65.1%) of patients were men and 35,264 (34.9%) were women [14].

An analysis of the Hospital Episode Statistics database in the United Kingdom revealed that the incidence of myocardial infarction per 100,000 population was 154 cases for men and 66 cases for women in 2010. Compared to 2002, the incidence rate decreased by 33% among men and 31% among women [15].

In Sweden, the incidence of myocardial infarction was estimated using data from the National Patient Registry, which recorded 320 cases per 100,000 population in 2019. The incidence per 100,000 population was 407 cases for men and 234 cases for women. By age, the incidence per 100,000 population was highest for those older than 85 years of age (2,079 cases) followed by those aged 80 years to 84 years (1,378 cases). Thereafter, the incidence increased with age. From 1991 to 2019, the overall incidence of myocardial infarction gradually decreased. In 1991, the incidence rate per 100,000 population was 634 cases; however, since then, it decreased steadily to 320 cases in 2019, which is approximately half of the earlier figure [16].

### Interpretation and discussion of hospitalization incidence

Based on the study of Kim et al. [4] from Korea, the age-standardized incidence of myocardial infarction for those who received inpatient treatment was 43.2 cases per 100,000 populations in 2016. The incidence rate in Korea was similar to or slightly higher than that in Japan, another East Asian country, and was very low compared to those in the United States, United Kingdom, and Sweden. One cause of these differences in the incidence rate may be racial differences. Analyses of the incidence and proportion of myocardial infarction by race in a single country [17-20] revealed that the incidence rate among Asians was lower than that among Whites or Blacks. In addition, the lower prevalence of major risk factors for myocardial infarction (high blood pressure, smoking, obesity, etc.) [7,20] among Asians compared to those among other races may also be the reason for the low incidence in Asia. However, looking at the trend from the past to the present, the trends in incidence in the United States [7,13,19], the United Kingdom [15], and Sweden [16] are gradually decreasing, whereas the incidence of myocardial infarction is gradually increasing in Korea. National support programs, such as educational programs, media campaigns, and public relations initiatives, are needed to actively decrease the incidence of myocardial infarction.

## PREVALENCE OF MYOCARDIAL INFARCTION

### Prevalence and trends

**Figure 3.**
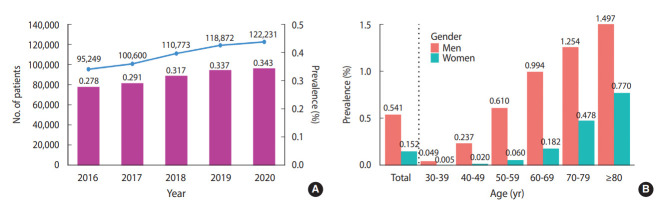
Prevalence of myocardial infarction and the number of myocardial infarction patients in Korea (A) by year from 2016 to 2020, and (B) by gender and age group. Source: Reproduced based on data from the Healthcare Bigdata Hub [1].

According to HIRA disease statistics, the number of patients with a diagnostic code of I21 (acute myocardial infarction) or I22 (subsequent myocardial infarction) was 122,231 in 2020, which translates to a prevalence of 0.343% of Korean adults aged 30 years and older. The annual prevalence was 0.278% in 2016, 0.291% in 2017, 0.317% in 2018, and 0.337% in 2019, indicating a gradual increase between 2016 and 2020 (Figure 3A).

### Prevalence by gender and age group

In 2020, the number of patients with myocardial infarction aged 30 years and older living in Korea was 121,959 [1]. Among them, 77.4% (n= 94,331) were men and 22.6% were women. The prevalence r of myocardial infarction among Koreans aged 30 years and older was 0.541% for men and 0.152% for women. By age, the highest prevalence of myocardial infarction was found among those aged 80 years and older, regardless of gender (Figure 3B).

### Prevalence and trends in other countries

Table 2 is showed the prevalence and trends in Korea, United States, and United Kingdom. In the United States, the prevalence of myocardial infarction was estimated from data collected by the NHANES [21]. From 2015 to 2018, the prevalence of myocardial infarction in the United States among those aged 20 years and older was 3.1% (95% confidence interval, 2.7 to 3.6), and it was 8.3% for men and 2.1% for women. By age, the prevalence rate was 0.4% for men and 0.4% for women aged 20 years to 39 years, 3.2% for men and 1.9% for women aged 40 years to 59 years, 12.6% for men and 4.5% for women aged 60 years to 79 years, and 15.8% for men and 8.7% for women 80 years and older.

In the United Kingdom, the prevalence of myocardial infarction was estimated using The Health Improvement Network database, which contains data covering approximately 6% of the population [22]. Analysis of this database found that the prevalence of myocardial infarction in the United Kingdom was 1.5% in 2017 (4.0% for men, 0.8% for women). By age, the highest prevalence was 6.1% among those aged 75 years and older, followed by 3.6% among those aged 65 years to 74 years, 2.0% among those aged 55 years to 64 years, and 0.7% among those aged 45 years to 54 years.

### Interpretation and discussion of prevalence

The prevalence of myocardial infarction in Korea, which was re-analyzed based on online open-source data, was lower than that in the United States and the United Kingdom. However, caution is needed when interpreting these results since there was a substantial difference in the prevalence (0.343 vs. 1.000%) between the 2 datasets from Korea, and the prevalence rates in the United States and United Kingdom (3.1 vs. 1.5%, respectively), which are Western countries, also differed by approximately 2 times. These differences in prevalence are likely due to the differences between the characteristics of the survey data and actual patient data. In other words, it could be due to the differences between the information obtained from the general public without accurate knowledge of the disease and the information provided by medical personnel. In this study, the prevalence from survey data was higher than that from patient data.

## MORTALITY FROM MYOCARDIAL INFARCTION

### Mortality and trends among the total population

**Figure 4.**
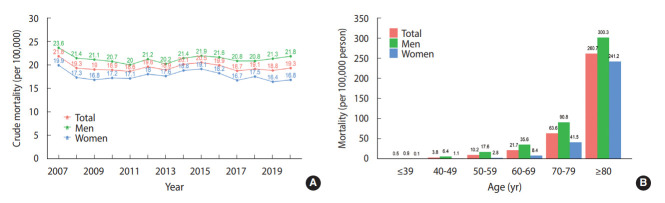
Crude mortality rate of acute myocardial infarction in Korea (A) by year from 2007 to 2020, and (B) by gender and age group. Source: Reproduced based on data from Statistics Korea [2].

An open-source report on yearly causes of death can be found on the Statistics Korea website [2]. According to these reports, the number of deaths due to acute myocardial infarction (code I21) in 2020 was 9,927, and the crude mortality rate was 19.3 deaths per 100,000 population (21.8 among men, 16.8 among women). Figure 4A shows the mortality rate in total and by gender from 2007 to 2020.

### Mortality by gender and age group

In 2020, the mortality rate for acute myocardial infarction increased with age, reaching 260.7 deaths per 100,000 population among those aged 80 years and older. The mortality rate was higher among men than among women, regardless of age group (Figure 4B).

### Mortality in other countries

**Figure 5.**
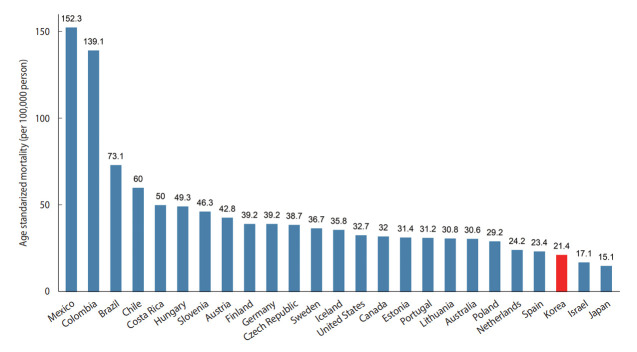
Age-standardized mortality rates of myocardial infarction (I21-I22) in Organization for Economic Cooperation and Development (OECD) countries in 2017. Source: Reproduced based on OECD Health Statistics data [23].

The OECD publishes the mortality rates of member countries according to the cause of death based on mortality data from the WHO [23]. Based on these statistics from 2017, the country with the highest mortality rate due to myocardial infarction (I21-I22) was Mexico, where the age-standardized mortality rate was 152.3 cases per 100,000 population, and the country with the lowest rate was Japan, where the age-standardized mortality rate was 15.1 cases per 100,000 population. Korea ranked 23rd among the 25 countries, with a mortality rate of 21.4 deaths per 100,000 population (Figure 5).

### Interpretation and discussion of mortality due to myocardial infarction

According to the 2017 OECD data [23], the mortality rate from myocardial infarction in Korea was lower than in other countries. In particular, the mortality rate in Korea was about 7 times lower than that in Mexico, which had the highest mortality rate. These differences between countries may be caused by multiple factors other than racial differences, including hospital structure, care processes, and organizational culture [24,25]. Some of this variation may also be explained by differences between countries concerning the minimum criteria required for certified doctors to specifically declare myocardial infarction as the underlying cause of death [26]. In other words, some countries are more conservative when confirming the cause of death as myocardial infarction when there is evidence of pathophysiology in the autopsy, while other countries take a more relaxed approach to death without requiring explicit clinical evidence. Therefore, rather than directly comparing mortality rates across countries, it is important to compare mortality trends from the past.

## STATISTICS OF ACUTE MYOCARDIAL INFARCTION USING INPATIENT REGISTRATION DATA

### Trends according to the type of myocardial infarction

Based on an analysis of KAMIR data [9] from 2018, 48.4% of all patients with myocardial infarction had STEMI and 51.6% had NSTEMI. In 2005, the proportion of patients with STEMI was 64.3%, which was higher than the proportion of patients with NSTEMI, but the proportion has gradually decreased since then. Even in the KRAMI data from the Regional Cardiocerebrovascular Disease Center, 42.4% of patients had STEMI and 57.6% had NSTEMI [9].

### Time from symptom onset to hospitalization/treatment

In the KAMIR data, the median time from symptom onset to treatment in patients diagnosed with myocardial infarction was 189 minutes in 2018, which reflects a marked decrease from 257 minutes in 2008 (16). The KRAMI calculates the median time from symptom onset to hospital arrival, which was 153 minutes for all patients in 2019 (119 minutes for STEMI, 196 minutes for NSTEMI) [10].

The National Emergency Medical Information Network database provides statistical information on the time from the onset of myocardial infarction to arrival in the emergency room [11]. According to these data, 37,988 patients experienced myocardial infarction in 2019, and a total of 20.7% of these patients arrived in less than 1 hour, 39.4% arrived within 2 hours, and 40.6% took 6 hours or longer to arrive. By gender, 22.9% of men and 15.7% of women arrived within less than 1 hour, whereas 37.1% of men and 48.6% of women arrived after more than 6 hours.

### Inpatient mortality

**Figure 6.**
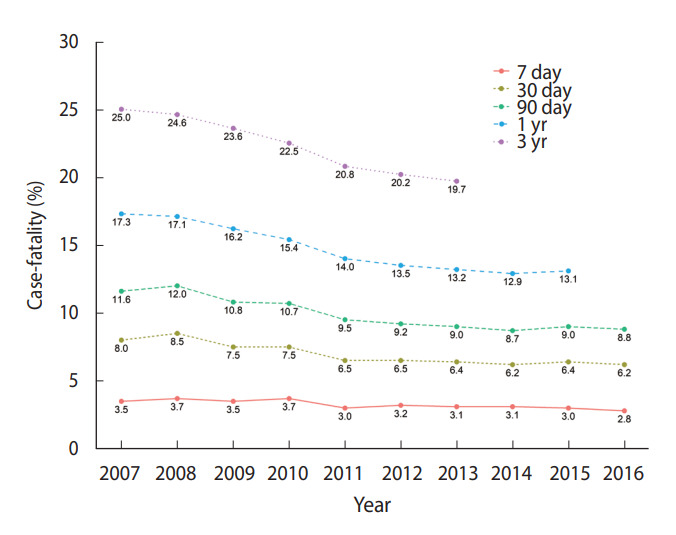
Case-fatality rate and trend of hospitalization with acute myocardial infarction from 2007 to 2016. Source: Adapted from Kim RB et al. J Korean Med Sci 2019;34:e322 [4].

According to a 2019 study [4] that analyzed data from the National Health Insurance Service and death data from Statistics Korea, the in-hospital mortality rate (death within 7 days of hospitalization) was 2.8% for patients with myocardial infarction who received inpatient treatment for more than 1 day in Korea in 2016. The 30-day mortality rate was 6.2%, the 90-day mortality rate was 8.8%, the 1-year mortality rate was 13.1%, and the 3-year mortality rate was 19.7%. Inpatient mortality has been decreasing for all indicators (Figure 6).

Based on the KAMIR registration data for hospitalized patients, the in-hospital mortality rate was 3.8% in 2018 [9], and 5.3% of these patients had STEMI and 2.4% had NSTEMI. The mortality rate within 1 year of discharge was 6.9% (7.9% STEMI, 6.1% NSTEMI). In the KRAMI data, the in-hospital mortality rate was 5.7% in 2019, whereas the 3-month mortality rate (including in-hospital deaths) was 7.3%. The mortality rate within 1 year, including in-hospital mortality, was 9.2% [10]. This is presumably because the KRAMI data include more patients who die in the acute stage.

According to data from the National Emergency Department Information System, which includes relatively detailed information on deaths from myocardial infarction in emergency rooms, 570 (1.5%) of 37,988 patients with acute myocardial infarction died in an emergency room in 2019, while 33,792 patients were hospitalized thereafter, and 2,911 (8.6%) died in hospital. Therefore, the in-hospital mortality rate after the onset of myocardial infarction was 9.2% [11].

### Comparison of mortality between hospitalized patients in other countries

Many other countries also maintain registries of patients with myocardial infarction. Most of these registry systems report inhospital mortality and 1-year inpatient mortality rates. The results are presented in Table 3.

### Interpretation and discussion of inpatient mortality from myocardial infarction

According to a study by Kim et al. [10], there were differences in in-hospital mortality and case-fatality (Unite States, ACTION-registry: 2.8% vs. Korea, NEDIS: 9.2%) among myocardial infarction patients by countries (Table 3). These differences are thought to be due to differences in patient characteristics included in each registry. In the case of NEDIS data, emergency department patients at all emergency medical center hospitals in Korea (regardless of whether PCI treatment is possible) are included, whereas, in the ACTION Registry, patients are registered only in hospitals capable of administering PCI. There were considered to be differences in in-hospital mortality. In addition, the differences in inhospital mortality in each registry may be caused by differences in the mean or median ages of enrolled patients. In the cases of the JAMIR and SWEDEHEART, since the patients included were older than those in other registries [10], in-hospital mortality (8.3 and 7.2%, respectively) was evaluated to be relatively high. In addition, in the case of the JAMIR, the proportion of STEMI patients was 79.7% [10], which was about twice as high as that of other registries, and in-hospital mortality is therefore believed to be higher in the JAMIR than in other registries. When comparing patients between registries from different countries, it is essential to evaluate the patient characteristics included in each registry.

## CONCLUSION

In this review, we summarized the epidemiological indicators of myocardial infarction from published articles and various data sources. Interpretation of these results requires understanding, and the characteristics of each dataset must be thoroughly considered. In particular, claims data from the National Health Insurance Service have the advantage of including the entire population of Korea; however, these data also have some disadvantages, including potential inaccuracy in diagnostic coding at the time when claims were made and a lack of information on the results of blood, clinical, and imaging tests. Mortality data from Statistics Korea have the advantage of identifying and annually publishing the causes of death across the entire population of Korea; however, they do not necessarily reflect the accurate causes of death since they are based on information from death certificates. Although comparisons of these data with data from other countries have been published, caution must be taken when making these comparisons due to differences in data collection and presentation methods between countries. Therefore, the epidemiological indicators of myocardial infarction described in this review should be interpreted with these advantages and limitations in mind.

Constructing a surveillance system for producing, monitoring, and evaluating epidemiological indicators of diseases at the national level is very important for disease prevention and management. Therefore, comprehensive support and research are needed at the national level to routinely produce accurate indicators related to myocardial infarction, which has a relatively large incidence, prevalence, or mortality.

### Ethics statement

This study was a review of published articles or opened data from the various Internet sources. The opened data did not include any personal information such as patient name, social security number, address, or phone number. So because there are no ethical issues, this study not needed institutional review board review.

## Figures and Tables

**Figure f7-epih-44-e2022057:**
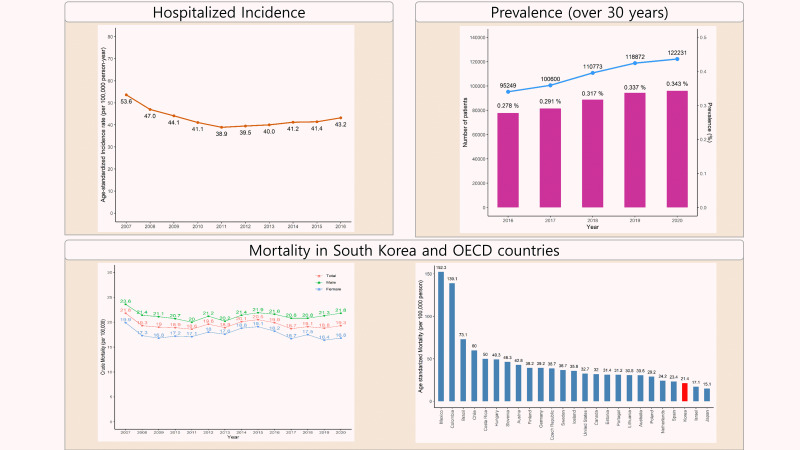


**Table 1.** Age-standardized incidence of acute myocardial infarction in several countries

Variables		Korea	Japan	USA	UK	Sweden
Year		2016	2016	2018	2018/19	2019
Incidence (per 100,000 population)						
	Total	43.2	40.7	261.0	151.5^1^	320.0
	Men	70.5^1^	67.1	358.0	200.4^1^	407.0
	Women	29.3^1^	17.1	176.0	104.8^1^	234.0
Trend of incidence		Slight increase	No change	Decrease	Decrease	Decrease
Data source		NHI claims data	Tokyo hospital data	Cohort data from Minnesota	Hospital patient data	National Patients Registry data
Definition of patients		ICD-10 code I21; more than 1 day of hospitalization for patients who had undergone lab, PCI, and antiplatelet tests; there was no I21 code for the 5 yr of data collection	AMI based on the universal definition from the ESC/ACC committee; admitted to a hospital in one of the 72 regions of Tokyo	ICD-10 code of I21 or I22; hospital or emergency room patients (aged 35 yr or older) at Minnesota state hospitals	ICD-10 code of I21 or I22; hospital or emergency room patients at all NHS hospitals in England	ICD-10 code of I21 or I22; hospital or emergency room patients (aged 20 yr or older) at all hospitals in Sweden

NHI, National Health Insurance; ICD-10, International Classification of Diseases, 10th edition; AMI, acute myocardial infarction; PCI, percutaneous coronary intervention; ESC/ACC, European Society of Cardiology/American College of Cardiology; NHS, National Health Service.

1 Crude incidence.

**Table 2.** Prevalence of myocardial infarction in several countries

Variables		Korea (NHI)	Korea (KNHANES)	USA	UK
Year		2020	2020	2018	2017
Prevalence (%)					
	Total	0.343	0.7	3.1	1.5
	Men	0.542	1.0	8.3	2.4
	Women	0.152	0.5	2.1	0.8
Trend of prevalence		Increase			
Source data		NHI claims data	KNHANES data [3]	NHANES data	Primary hospital patient data
Definition of patients		ICD-10 code of I21 or I22; inpatient or outpatient (aged 30 yr or older) of all hospitals in Korea	Individuals (aged 30 yr or older) who responded “yes” to the survey question, “Do you currently have a disease related to myocardial infarction?”	Individuals (aged 20 yr or older) who responded “yes” to the survey question, “Has a doctor or other health professional ever told you that you had a heart attack or MI [myocardial infarction]?”	Myocardial infarction patients with symptoms who received treatment at primary health care centers

NHI, National Health Insurance; KNHANES, Korea National Health and Nutrition Examination Survey; NHANES, National Health and Nutrition Examination Survey; ICD-10, International Classification of Diseases, 10th edition.

**Table 3.** In-hospital and 1-year case-fatality of acute myocardial infarction patients in registries by countries

Variables	KRAMI-RCC	KAMIR-NIH	NEDIS	JAMIR	CAMI registry	SWEDEHEART	AMIS	ACTION-Registry
Country	Korea	Korea	Korea	Japan	China	Sweden	Swiss	USA
Period of registry	Jul 2016-Jun 2019	Nov 2011-Oct 2015	2019	Jan 2011-Dec 2013	Jan 2013-Sep 2014	Jan 2006-Dec 2013	Jan 1997-Jun. 2009	Jan 2007-Dec 2014
No. of patients	11,758	13,624	37,988	20,462	26,103	289,699	31,010	322,523
In-hospital case-fatality (%)	5.6	3.9	9.2	8.3	4.9 (STEMI)/4.2 (NSTEMI)	7.2	6.8	2.8
1-yr case-fatality (%)	4.4	4.3		-	-	12.0	4.0	-

KRAMI-RCC, Korean Registry of Acute Myocardial Infarction for Regional Cardiocerebrovascular Centers; KRAMIR-NIH, Korea Acute Myocardial Infarction Registry - National Institutes of Health; NEDIS, National Emergency Department Information System; JAMIR, Japanese Acute Myocardial Infarction Registry; CAMI, China Acute Myocardial Infarction; SWEDEHEART, Swedish Heart Intensive Care Admissions; AMIS, Acute Myocardial Infarction in Switzerland; ACTION-Registry, Acute Coronary Treatment and Intervention Outcomes Network Registry; STEMI, ST-segment elevation myocardial infarction; NSTEMI, non-ST-segment elevation myocardial infarction. Source: Adapted from Kim RB et al. J Clin Med 2021;10:498 [10].
